# Design and Synthesis of Functional Silane-Based Silicone Resin and Application in Low-Temperature Curing Silver Conductive Inks

**DOI:** 10.3390/nano13061137

**Published:** 2023-03-22

**Authors:** Zhiqiang Tang, Yanxia Liu, Yagang Zhang, Zicai Sun, Weidong Huang, Zhikai Chen, Xiaoli Jiang, Lin Zhao

**Affiliations:** 1 School of Materials and Energy, University of Electronic Science and Technology of China, Chengdu 611731, China; 202021030330@std.uestc.edu.cn (Z.T.); liuyanxia100@uestc.edu.cn (Y.L.); 202121030326@std.uestc.edu.cn (W.H.); 202021030329@std.uestc.edu.cn (Z.C.); jiangxl@std.uestc.edu.cn (X.J.); zhaolin316@uestc.edu.cn (L.Z.); 2 Dongguan Yimei Material Technology Co., Ltd., Dongguan 523000, China

**Keywords:** functional silicon monomers, silicone resin, low-temperature curing, silver conductive inks

## Abstract

In the field of flexible electronics manufacturing, inkjet printing technology is a research hotspot, and it is key to developing low-temperature curing conductive inks that meet printing requirements and have suitable functions. Herein, methylphenylamino silicon oil (N75) and epoxy-modified silicon oil (SE35) were successfully synthesized through functional silicon monomers, and they were used to prepare silicone resin 1030H with nano SiO_2_. 1030H silicone resin was used as the resin binder for silver conductive ink. The silver conductive ink we prepared with 1030H has good dispersion performance with a particle size of 50–100 nm, as well as good storage stability and excellent adhesion. Additionally, the printing performance and conductivity of the silver conductive ink prepared with n,n-dimethylformamide (DMF): proprylene glycol monomethyl ether (PM) (1:1) as solvent are better than those of the silver conductive ink prepared by DMF and PM solvent. Cured at a low temperature of 160 °C, the resistivity of 1030H-Ag-82%-3 conductive ink is 6.87 × 10^−6^ Ω·m, and that of 1030H-Ag-92%-3 conductive ink is 0.564 × 10^−6^ Ω·m, so the low-temperature curing silver conductive ink has high conductivity. The low-temperature curing silver conductive ink we prepared meets the printing requirements and has potential for practical applications.

## 1. Introduction

Over the last decade, there has been growing interest in printed electronics for the creation of flexible electronic circuits and devices using printing technology [1,2,3,4,5,6]. This is because printing technology can offer benefits such as cost-effectiveness, reduced waste generation, and eco-friendly printing techniques. Compared to traditional methods of producing electronic devices, such as photolithography [7], vacuum deposition [8], and electroless plating processes [9], printing technology offers a more streamlined, efficient, and environmentally conscious alternative. These traditional methods often require multiple steps, expensive equipment, and the use of harmful chemicals that produce significant amounts of waste [10,11].

Printed electronics have been applied to sensors [12,13], transistors [14,15], solar cells [16,17], radio frequency identification [18], and photovoltaics [19]. One of the notable properties of printed electronics is their ability to be flexible and lightweight, making them well-suited for wearable devices [20,21]. Among the key components of wearable electronic devices, conductors are considered essential for establishing connections between different components. Consequently, there is a need to develop suitable conductive inks for the fabrication of conductors on flexible substrates such as circuit microelectrodes [22,23,24].

Conductive inks based on silver [25,26,27], copper [28,29], carbon nanotubes [30], and conducting polymers [31] have garnered significant interest. The carbon series and metal salts have been found to exhibit unsatisfactory conductivity [32], leading to a surge in research into conductive inks based on metal nanoparticles. Metal particles such as Au, Ag, and Cu are commonly used conductive materials in printed electronics [33]. Among them, Ag is the most commonly used due to its high conductivity, anti-oxidation properties, and stability at ambient temperature [34]. Therefore, silver conductive inks are extremely attractive, and, in addition, Ag can be incorporated into inks that adhere better to substrates than other metals [35]. Until now, silver conductive inks have been utilized in stretchable devices, flexible display screens, wearable sensors, etc [36].

The quality and performance of silver conductive inks are generally dependent on the components used in their preparation. The application of silver conductive ink is limited by many factors, such as a complex ink preparation process, as well as potential conflicts between ink stability and solid content. Low solid content decreases the thickness of printed tracks, leading to high pattern resistance. Additionally, the sintering temperature of commercial silver conductive ink is high (higher than 200 °C) [37]. As a result, polymeric stabilizers are often used to improve the stability of high-solid-content conductive ink and reduce the curing temperature [38]. With the addition of the resin binding phase, new challenges have emerged, such as poor conductivity and easy agglomeration of silver conductive inks [39]. There is still much room for improvement in current silver conductive ink printing technology. Htwe et al. [40] prepared graphene/Ag conductive inks by mixing graphene and Ag, which exhibited better stability, wettability, and electrical conductivity compared to those graphene and Ag conductive inks. Under 50% tensile and 100 bending times, the printed hybrid conductive traces exhibited a 4% and 3% drop in conductivity, respectively. Zhang et al. [27] fabricated the high conductive and stable Ag pattern on a flexible photo paper substrate via the addition of Polymer vinyl acetate (PVAc) in the ink solvents, which played an important role in controlling the patterns’ adhesion. However, the addition of PVAc in Ag conductive ink also results in decreasing the patterns’ conductivity. It is well known that silicone resin can be cured at a lower temperature and has excellent adhesion [41]. We can combine the performance of silicone resin with silver conductive ink.

In the field of flexible electronics manufacturing, inkjet printing technology has always been a research hotspot, and it is key to developing low-temperature curing conductive inks that meet printing requirements and have suitable functions. In this work, methylphenylamino silicon oil (N75) and epoxy-modified silicon oil (SE35) were successfully synthesized through functional silicon monomers, and 1030H silicone resin was prepared as the resin binder for low-temperature curing silver conductive ink. The addition of 1030H silicone resin suppressed the aggregation of nano silver particles, making the prepared low-temperature curing silver conductive ink have good dispersion performance with a particle size of 50–100 nm, as well as good storage stability and excellent adhesion. In addition, we discussed the effect of different solvents on the performance of silver conductive ink. Among them, the optimal solvent is n,n-dimethylformamide (DMF): proprylene glycol monomethyl ether (PM) (1:1). The silver conductive ink prepared with the solvent (DMF:PM (1:1)) not only meets the printing requirements but also can be cured at a relatively low temperature, with a curing temperature below 200 °C. The pattern printed with silver conductive ink has high conductivity. The low-temperature curing silver conductive ink we prepared has potential for the fabrication of conductors on flexible substrates as circuit microelectrodes.

## 2. Materials and Methods

### 2.1. Materials

Phenyltrimethoxysilane (FEOS), diethoxydimethylsilane (DMDES), diphenyldimethoxysilane (DMDPS), (3-aminopropyl)trimethoxysilane (APTMS), (3-aminopropyl)dimethoxymethylsilane (APDMS), tetraethyl orthosilicate (TEOS), hexamethyldisiloxane (MM), 3-glycidyloxypropyltrimethoxysilane (GOPTS), nano silica (SiO_2_), N,N-dimethylformamide (DMF), and proprylene glycol monomethyl ether (PM) were bought from Aladdin, China, and used as obtained. Nano silver powder was bought from BroadCON, China, and used as obtained.

### 2.2. Synthesis of Methylphenylamino Silicone Oil (N75)

**Figure 1:**
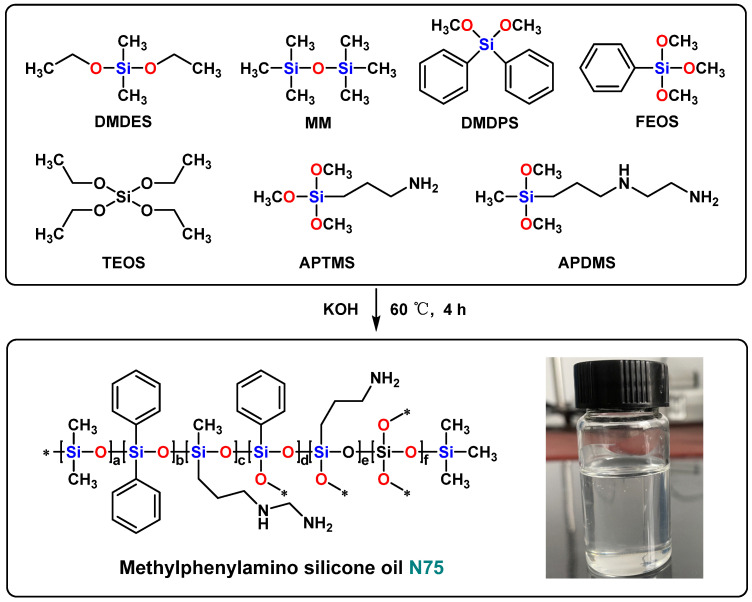
Synthesis of methylphenylamino silicone oil (N75).

Under the protection of nitrogen gas, 7.4 g of DMDES, 12.6 g of DMDPS, 1.76 g of APTMS, 2.9 g of APDMS, 3.16 g of FEOS, 1.03 g of TEOS, and 4.54 g of MM were charged in a 250 mL flask equipped with a stirrer, reflux condenser, and constant pressure titration funnel. Then, 0.04 g potassium hydroxide was dissolved in 7 g of ethanol–water solution (mass ratio 1:1), stirred and added to the flask. Under the catalysis of potassium hydroxide, the mixture was stirred at 60 °C for 4 h. The system was then slowly heated to 130 °C to remove the generated alcohol and excess water, resulting in a transparent viscous oil. A certain amount of cyclohexane was added to the oil and then 732 cation exchange resin was used to remove the strong base catalyst from the reaction system. The resulting liquid was filtered and distilled to remove the cyclohexane at 140 °C. H_2_O was removed under vacuum to obtain methylphenylamino silicone oil (N75). The hydrogen value of the synthesized N75 was 0.68 mol/100 g, and the synthetic process is shown in Figure 1.

### 2.3. Synthesis of Epoxy-Modified Silicone Oil (SE35)

**Figure 2:**
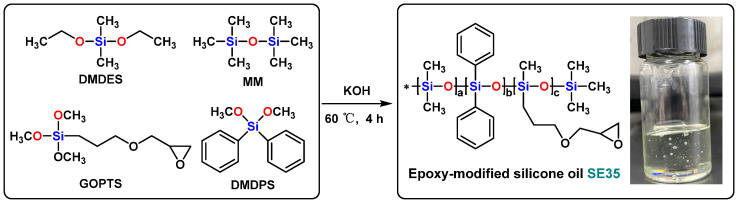
Synthesis of epoxy-modified silicone oil (SE35).

Under the protection of nitrogen gas, 5.55 g of DMDES, 9.45 g of DMDPS, 1.76 g of GOPTS, and 2.27 g of MM were charged in a 250 mL flask equipped with a stirrer, reflux condenser, and constant pressure titration funnel. Then, 0.02 g of potassium hydroxide was dissolved in 3.5 g of ethanol–water solution (mass ratio 1:1), stirred and added to the flask. Under the catalysis of potassium hydroxide, the mixture was stirred at 60 °C for 4 h. The system was then slowly heated to 130 °C to remove the generated alcohol and excess water, resulting in a transparent viscous oil. A certain amount of cyclohexane was added to the oil and then 732 cation exchange resin was used to remove the strong base catalyst from the reaction system. The resulting liquid was filtered and distilled to remove the cyclohexane at 140 °C. H_2_O was removed under vacuum to obtain epoxy-modified silicone oil (SE35). The epoxy value of the synthesized SE35 was 0.35 mol/100 g, and the synthetic process is shown in Figure 2.

### 2.4. Preparation of 1030H Silicone Resin

**Figure 3:**
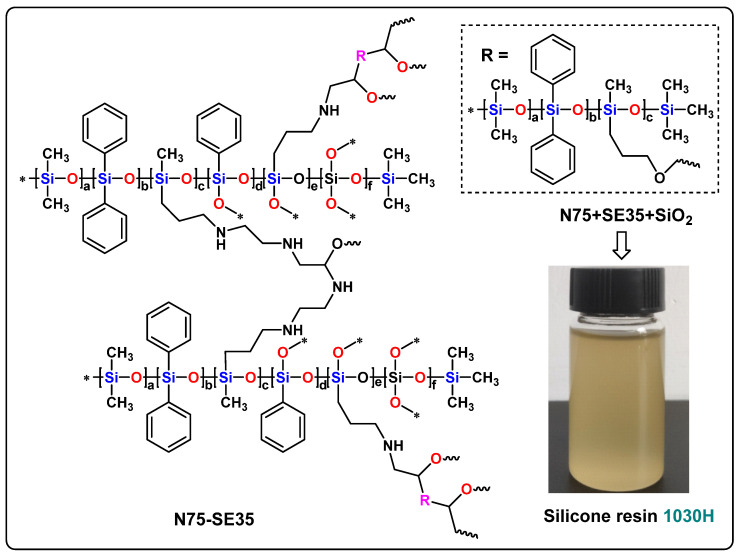
Reaction product of N75 and SE35.

5.00 g of methylphenylamino silicone oil (N75), 7.42 g epoxy-modified silicone oil (SE35) (The ratio of epoxy value to hydrogen value is 1 to 0.97. The slight excess of epoxy was added to ensure complete reaction of active hydrogen, which is extremely detrimental to electronic products), and 14.71 g of DMF solvent were added to a beaker and stirred for 1 h. Then, 2 w% of nano SiO_2_ was added and stirred for 1 h to obtain 1030H silicone resin. The 1030H silicone resin undergoes a curing reaction at 120 °C, and the reaction product of N75 and SE35 is shown in Figure 3.

### 2.5. Preparation of Silver Conductive Ink

The silver conductive ink was prepared by using 1030H silicone resin as the resin carrier and nano silver powder as the conductive filler, which was uniformly dispersed in the solvent (DMF, PM, DMF:PM (1:1)). The formulations for the silver conductive ink are shown in Table 1. The nano silver powder was dispersed and diluted to 70% using the solvent, and the dispersion machine was used to stir the mixture at high speed to uniformly disperse the nano silver powder in the solvent. Then, the 1030H silicone resin was slowly added to the dispersed nano silver powder solution, and the remaining solvent was added to the solution. The entire process was carried out under high-speed stirring. The resulting solution was placed in a shaking disperser for 2 h to obtain a uniformly dispersed silver conductive ink.

**Figure 4:**
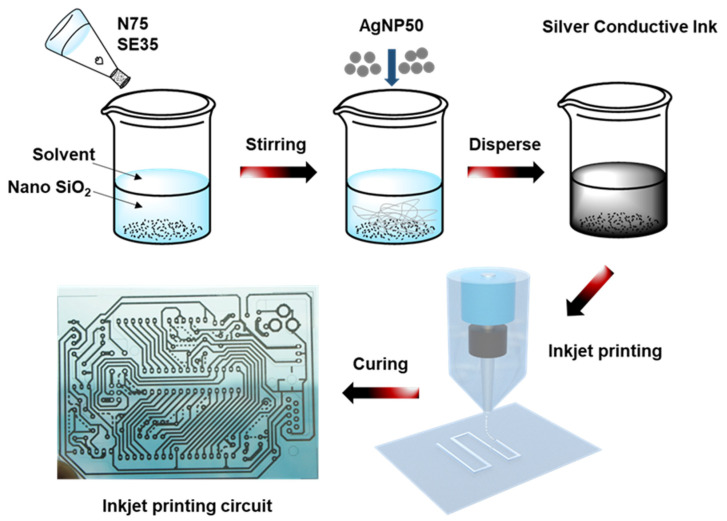
Preparation of silver conductive ink.

The prepared silver conductive ink was printed on a 50 μm thick polyethylene terephthalate (PET) film using an inkjet printing process. The Micro-Electronic Printer purchased from Shanghai CCI Instrument Co., Ltd., Shanghai, China, (including an ink box with 16 nozzles with diameters of 20 µm arranged in a row) was used to print silver conductive patterns. The number of nozzles used for inkjet printing can be controlled using Bits Assembler (software for controlling Micro-Electronic Printer). In addition, the inkjet process of each nozzle is controlled via each piece of piezoelectric ceramics. The pattern printed had a thickness of 10 μm, a length of 10 cm, and a width of 1 mm. The printed silver conductive ink was then cured to ensure that the conductive ink was stably attached to the film. The curing conditions were drying at 90 °C to volatilize solvent to achieve semi-curing, hot pressing at 145 °C for 5 min in a film laminator, and then curing at 160 °C for 2 h to obtain printed and cured conductive patterns. The preparation and printing process of silver conductive ink is shown in Figure 4.

### 2.6. Characterization

Fourier transform infrared spectra (FT-IR) were obtained using a Niolet iN10 instrument with an ATR attachment, operating over 400–4000 cm^−1^ at ambient temperature. The nuclear magnetic resonance (NMR) measurements were carried out on an Avance NEO 600 (Bruker) at 500.13 MHz (^1^H). The spectra were referenced to the solvent signal (CDCl_3_: δ (^1^H) = 7.26 ppm). Thermogravimetric analysis (TGA) data were recorded on a Mettler tga 2 instrument over the range of 25 to 800 °C, with a heating rate of 5 °C min^−1^ under a flow of N_2_ (20 mL min^−1^). SEM and EDX were acquired on a Schottky Field Emission Scanning Electron Microscope ZEISS MERLIN Compact operating in variable pressure mode with an accelerating voltage of 15 keV.

## 3. Results and Discussion

### 3.1. Characterization of N75 and SE35

Methylphenylamino silicone oil (N75) was synthesized by the hydrolysis and co-condensation reaction under the catalysis of Potassium hydroxide (Figure 1). Epoxy-modified silicone oil (SE35) was synthesized by the hydrolysis and co-condensation reaction under the catalysis of potassium hydroxide (Figure 2). N75-SE35 was synthesized by cross-linking and curing N75 and SE35 (Figure 3).

#### 3.1.1. FT-IR

**Figure 5:**
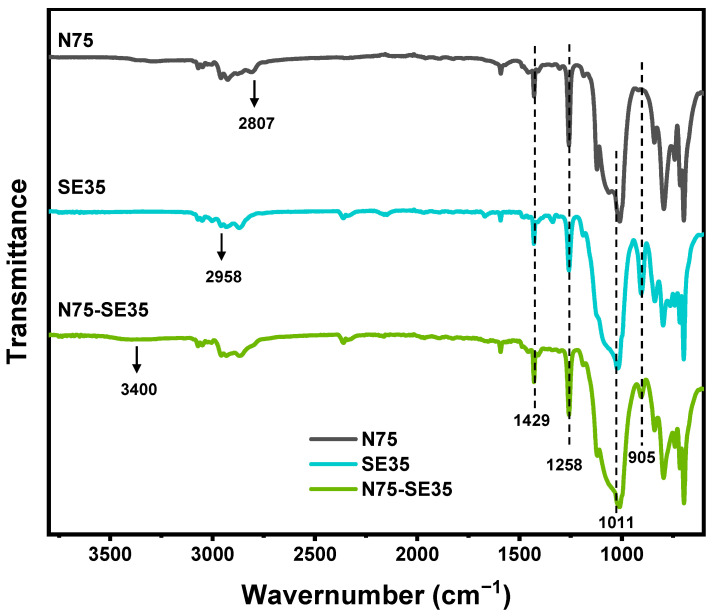
The FT-IR spectra of N75, SE35, and N75-SE35.

To confirm the occurrence of the reaction to synthesize N75-SE35, FT-IR was carried out to compare the chemical structures of N75, SE35, N75-SE35, and the spectra are shown in Figure 5. In the spectrum of N75, the peak at 3100–3000 cm^−1^ was assigned to the characteristic stretching vibration of C-H, corresponding to the benzene ring. The peak at 2958 cm^−1^ was attributed to the saturated C-H stretching vibration, including -CH_2_- and -CH_3_. while the peaks at 1429 cm^−1^ and 1258 cm^−1^ were the characteristic absorption peaks of -Si-CH_2_- and -Si-CH_3_, respectively [42]. The peak at 1011 cm^−1^ was the characteristic absorption peak of the organic silicon framework Si-O-Si [43]. The FT-IR spectrum of N75 exhibited a broad peak around 3400 cm^−1^, which was attributed to the N-H characteristic absorption peak, and a peak at 2807 cm^−1^, which was attributed to the characteristic absorption peak of C-NH [44]. In the spectrum of SE35, a strong absorption peak was observed at 905 cm^−1^, which was attributed to the characteristic absorption peak of the epoxy group in SE35. In summary, N75 and SE35 were successfully synthesized. In the spectrum of N75-SE35, its FT-IR spectrum contained characteristic absorption peaks of both N75 and SE35. It can be clearly seen that the intensity of the characteristic absorption peak of the epoxy group at 905 cm^−1^ was significantly reduced, indicating that N75 and SE35 have undergone the curing reaction to generate N75-SE35.

#### 3.1.2. ^1^H-NMR

**Figure 6:**
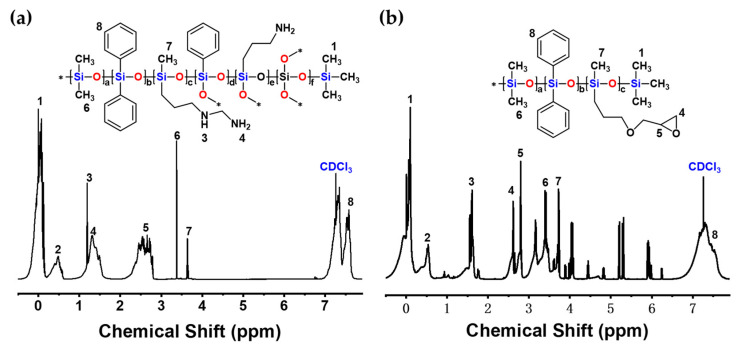
The ^1^H-NMR spectra of N75 (**a**) and SE35 (**b**).

The ^1^H-NMR spectra of N75 and SE35 are shown in Figure 6. For the ^1^H-NMR spectrum of N75, the signals at 0.09 ppm and 3.37 ppm were attributed to (CH_3_)_3_SiO_1/2_ and (CH_3_)_2_SiO, respectively. The signals at 1.19 ppm and 1.37 ppm were attributed to N-H from NH_2_(CH_2_)_2_NH(CH_2_)_3_Si. The signal at 7.55 ppm was typically attributed to C-H from the benzene ring. The signals at 0.48 ppm, 2.67 ppm, and 3.64 ppm were assigned to NH_2_(CH_2_)_2_NH, NH(CH_2_)_3_Si, and CH_3_SiO_3/2_, respectively. For the ^1^H-NMR spectrum of SE35, the signal at 0.09 ppm was attributed to the -CH_3_ of Si(CH_3_)_3_. The signals at 2.62 ppm and 2.80 ppm were attributed to C-H from the epoxy group. The signals at 0.52 ppm, 1.58 ppm, 3.41 ppm, and 3.71 ppm were assigned to CH_2_OCH_2_, CH_2_CH_2_O, CH_3_Si, and (CH_3_)_2_SiO, respectively [45,46,47]. Based on the above ^1^H-NMR and FT-IR analysis, the chemical structure of N75 and SE35 were in good agreement with the anticipated structure.

### 3.2. Properties of Silver Conductive Inks

Conductive ink is a key material in electronic printing technology. After printing, the ink functions like a wire by providing a conductive connection. The quality of flexible electronic devices is influenced by the properties of the conductive ink, such as its dispersion uniformity, conductivity after printing, and adhesion to the substrate material. Silver conductive inks with different formulations exhibited various performance characteristics after printing (Table 1).

#### 3.2.1. Particle Size

Inkjet printing requires a specific particle size for conductive ink particles, as particles that are too large will clog the nozzles. However, in silver conductive ink, the silver nanoparticles tend to aggregate, leading to particles that are too large. Thus, suppressing the aggregation of silver nanoparticles in silver conductive inks is a challenging issue. In this study, 1030H resin was designed and synthesized to suppress the aggregation of silver nanoparticles.

**Figure 7:**
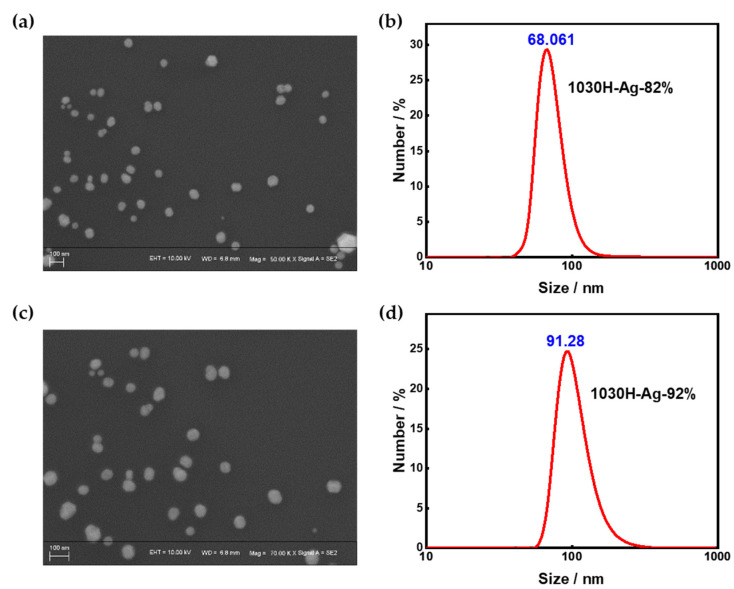
(**a**) SEM of 1030H-Ag-82% conductive ink; (**b**) Particle size distribution chart of 1030H-1030H-Ag-82% conductive ink; (**c**) SEM of 1030H-Ag-92% conductive ink; (**d**) Particle size distribution chart of 1030H-Ag-92% conductive ink.

Figure 7a,b are the SEM image and particle size distribution of 1030H-Ag-82% conductive ink. The particle size distribution of 1030H-Ag-82% conductive ink was uniform, and the peak value of the particle size was 68.061 nm. The silver nanoparticles did not aggregate, which is due to the addition of 1030H resin to the conductive ink. The electrostatic interaction between N in 1030H resin and silver nanoparticles makes it difficult for the silver nanoparticles to aggregate.

Figure 7c,d are the SEM image and particle size distribution of 1030H-Ag-92% conductive ink. The particle size distribution of 1030H-Ag-92% conductive ink was also uniform, with a peak particle size 91.28 nm higher than that of 1030H-Ag-82% conductive ink. This is because the proportion of 1030H resin in 1030H-Ag-82% conductive ink is higher, and the effect of suppressing the aggregation of silver nanoparticles is more obvious.

#### 3.2.2. Storage Stability

**Figure 8:**
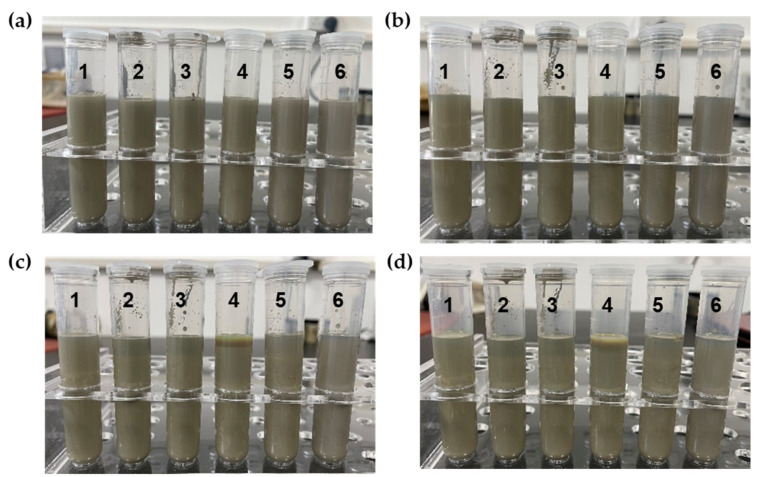
Storage stability of silver conductive ink (numbers 1–6 are 1030H-Ag-82%, 1030H-Ag-84%, 1030H-Ag-86%, 1030H-Ag-88%, 1030H-Ag-90%, and 1030H-Ag-92%, respectively). (**a**) 1d; (**b**) 30d; (**c**) 45d; (**d**) 60d.

The conductive filler in silver conductive ink is silver nanoparticles. Compared to the density of solvents and resins, silver nanoparticles have a high density (10.49 g·cm^−3^) and tend to agglomerate in conductive inks. Silver conductive ink may experience varying degrees of delamination after long-term transportation and storage. Therefore, silver needs to have certain storage stability after uniform dispersion in conductive ink. To explore the storage stability of the prepared silver conductive ink, silver conductive inks with different formulations were stored at room temperature (25 °C) for 60 days, and the delaminating phenomenon was observed and recorded by taking photographs, as shown in Figure 8.

After 30 days of storage, the silver conductive ink remained stable without delamination (Figure 8b). However, after 45 days of storage, some formulations of silver conductive inks showed slight delamination, and the degree of delamination increased with the increase in silver content (Figure 8c). The conductive ink with 82% Ag showed no delamination. This is because the 1030H-Ag-82% conductive ink contains a higher proportion of 1030H silicone resin, which inhibits the agglomeration of silver nanoparticles. After 60 days of storage, the conductive ink with high silver content only slightly delaminated, as shown in Figure 7d (4–6), and it could continue to be used normally after shaking well before use. Therefore, the silver conductive ink we prepared has excellent storage stability and can meet the requirements of long-term transportation and storage. The excellent properties of silver conductive ink benefit from the addition of 1030H silicone resin.

#### 3.2.3. Adhesion

Adhesion performance is evaluated according to ASTM D3359 [48]. It is a standard test method for measuring adhesion by tape test. This test method is used to determine the adhesion of coatings to substrates. Following the ASTM (D3359) tape adhesion test, grid lines with 1 mm spacing were carved into the coating on glass by a sharp blade. Then, 3M 600 tape was applied to the coating surface under a 2 Kg weight for 90 s, then the tape was quickly removed at a 180-degree angle. The amount of coating removed from the substrate was then visually assessed using a rating scale. The rating scale used in ASTM D3359 ranges from 0B (more than 65% adhesion loss) to 5B (no adhesion loss). The silver conductive inks demonstrated excellent adhesion to PET substrates. In fact, silver conductive inks prepared without 1030H silicone resin showed poor adhesion on smooth PET substrates. The adhesion of silver conductive ink increased with the content of 1030H silicone resin. When the silver content increased from 82% to 92%, the adhesion classification decreased from 5B to 4B. The 1030H-Ag-82% conductive ink had the highest adhesion classification—5B on PET—and the adhesion classification of the 1030H-Ag-92% conductive ink with less 1030H silicone resin was 4B (Table 1).

#### 3.2.4. Pencil Hardness

The pencil hardness of printed patterns was evaluated according to ASTM D3359 [49]. The hardness of the printed pattern was measured by the trolley method. The printed pattern was placed horizontally, the pencil was fixed downward at an angle of 45°, and the pen tip applied a load of 750 g on the surface of the pattern by the mass of the trolley and the counterweight. The trolley was pushed horizontally across the printed pattern at a constant speed by the hand push method, and the hardness of the pencil was gradually increased until permanent indentations or visible scratches appeared on the surface of the printed pattern. For this test, pencils of increasing hardness value were moved over the surface in a precisely defined way until one lead damages the surface. Surface hardness is defined by the hardest pencil grade which just fails to damage the surface. Grading pencils come in an assortment of both hard and soft. The hardest is 9H, followed by 8H, 7H, 6H, 5H, 4H, 3H, 2H, and H. F is the middle of the hardness scale. The hardness is then HB, B, 2B, 3B, 4B, 5B, 6B, 7B, 8B, and 9B, which is the softest. Conductive inks without 1030H silicone had poor pencil hardness and little adhesion on smooth PET substrates. After the addition of the 1030H silicone resin, the pencil hardness of the printed pattern was also improved to 2H or higher (Table 1). The pencil hardness ensured that the printed pattern did not leave scratches when rubbed. This is mainly due to the excellent performance of 1030H silicone resin.

#### 3.2.5. Conductivity

The conductivity of the silver conductive ink is the most important performance characteristic that directly determines whether the conductive ink is qualified. The resistivity of the patterns printed with different formulations of the silver conductive ink is shown in Table 1. As the silver content in the conductive inks increase, the resistivity decreases, and the conductivity of the silver conductive inks improved. The resistivity trend is obvious when the silver content increased from 1030H-Ag-78% to 1030H-Ag-82%. The resistivity decreased from 934 × 10^−6^ Ω·m to 28.1 × 10^−6^ Ω·m. This unique phenomenon is called the percolation threshold theory in conductive inks. When the content of conductive filler in the conductive ink reaches a certain amount, the resistivity drops rapidly and the conductivity increases greatly. As the silver content in the conductive inks continues to increase, the resistivity trend slows down. When the silver content increased from 82% to 92%, the resistivity only decreased from 28.1 × 10^−6^ Ω·m to 1.43 × 10^−6^ Ω·m. Therefore, further increasing the silver content did not have a significant effect on the conductivity performance. The results indicated that the use of 1030H silicone resin as the resin binder phase of the silver conductive ink not only provides excellent adhesion performance, but also good conductivity performance.

#### 3.2.6. Elemental Composition

**Figure 9:**
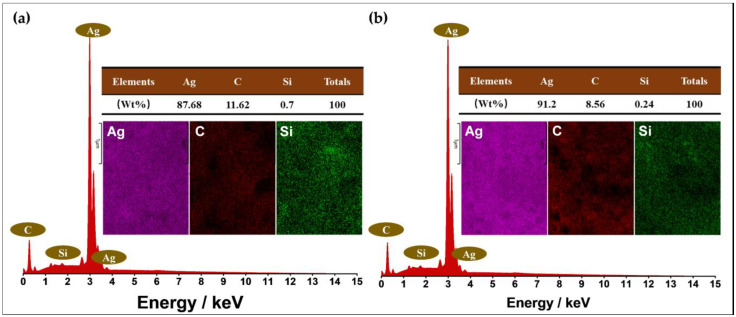
The EDS spectra of Ag conductive patterns. (**a**) 1030H-Ag-82% conductive ink; (**b**) 1030H-Ag-92% conductive ink.

The silver content in the silver conductive ink directly determines the conductivity of the printed pattern, and the amount of silver directly determines the cost of the silver conductive ink. The elemental compositions of 1030H-Ag-82% conductive ink and 1030H-Ag-92% conductive ink printed patterns were characterized by EDS, as shown in Figure 9. It can be seen that the proportion of silver in 1030H-Ag-82% conductive ink is 87.68%, and the proportions of C and Si are 11.62% and 0.7%, respectively (Figure 9a). The printed pattern of 1030H-Ag-82% conductive ink has a resistivity of 28.1 × 10^−6^ Ω·m (Table 1) after sintering. The proportion of silver in 1030H-Ag-92% conductive ink increased to 91.2%, while the proportions of C and Si decreased to 8.56% and 0.24%, respectively (Figure 9b). The printed pattern of 1030H-Ag-92% conductive ink has a resistivity of 1.43 × 10^−6^ Ω·m (Table 1) after sintering. Obviously, the higher the proportion of silver, the lower the resistivity of the printed pattern after sintering, and the better the conductivity. Adding an appropriate amount of 1030H silicone resin to the silver conductive ink can not only improve the printing performance of the conductive ink, but also reduces the proportion of silver, thereby reducing the cost of the silver conductive ink.

### 3.3. Effect of Solvent on Properties of Silver Conductive Ink

Solvent is an important component of silver conductive ink. It can not only dissolve the resin binder to disperse the conductive filler more effectively, but can also adjust the viscosity of the conductive ink and affect its curing temperature. Usually, we dissolve the resin binder in the solvent first, as the resin carrier of the conductive filler, to disperse the conductive filler. The solvents for different resins may vary slightly, and there are also many types of solvents for the same resin. Therefore, the resin carriers prepared with different solvents may have different characteristics, and the resulting silver conductive ink may also have different performance characteristics.

In the previous section, we discussed the performance of silver conductive ink using DMF as solvent. In this section, we discuss the influence of different solvents on the performance of silver conductive ink. We selected three solvents, which were DMF solvent, PM solvent, and a solvent system consisting of DMF and PM in equal proportions. We also selected two different formulations of conductive ink with different silver contents, which were 1030H-Ag-82% conductive ink and 1030H-Ag-92% conductive ink, as shown in Table 2.

#### 3.3.1. Surface Tension

**Figure 10:**
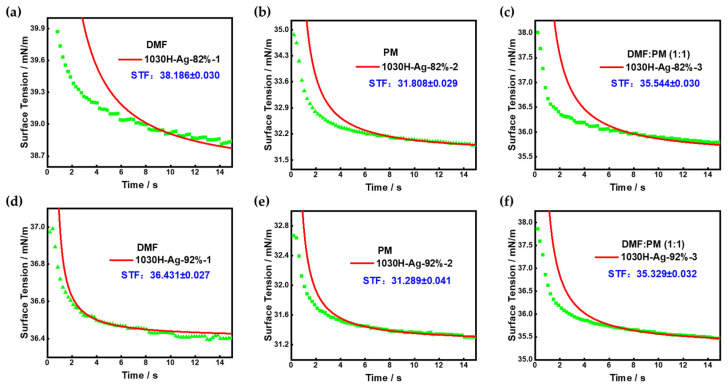
Surface tension of silver conductive ink in different solvent systems. (**a**) 1030H-Ag-82%-1 conductive ink; (**b**) 1030H-Ag-82%-2 conductive ink; (**c**) 1030H-Ag-82%-3 conductive ink; (**d**) 1030H-Ag-92%-1 conductive ink; (**e**) 1030H-Ag-92%-2 conductive ink; (**f**) 1030H-Ag-92%-3 conductive ink.

The surface tension of silver conductive ink is of great importance in inkjet printing. The surface tension of conductive ink should not be too low, as it will come into contact with air easily, resulting in bubbles and affecting printing quality. The surface tension of conductive ink should not be too high either, as high surface tension can lead to uneven ink coating on the substrate during the printing process, resulting in breaks or turbidity. Therefore, we used solvents to adjust the surface tension of conductive inks to ensure printing quality. The surface tensions of the 1030H-Ag-82% conductive ink and 1030H-Ag-92% conductive ink prepared using different solvents were tested, and the results are shown in Figure 10.

Under the same solvent system conditions, the surface tension of 1030H-Ag-82% conductive ink was higher than that of 1030H-Ag-92% conductive ink, which is because the proportion of 1030H silicone resin in 1030H-Ag-82% conductive ink is higher. The surface tension of the conductive inks fabricated by DMF as the dispersing solvent, 1030H-Ag-82%-1 conductive ink (Figure 10a) and 1030H-Ag-92%-1 conductive ink (Figure 10d), was 38.17 mN/m and 36.43 mN/m, respectively, which is higher than the surface tension of the conductive ink prepared by the other two solvents. The surface tension of the conductive ink fabricated with PM as solvent was the lowest, about 31 mN/m (Figure 10b,e). The required surface tension for printing inks is in the range of 30–40 mN/m, with an optimal surface tension of 35 mN/m. Therefore, a mixed solvent of DMF and PM can be used to adjust the surface tension of the ink. Figure 10c,f show that the surface tension of silver conductive ink 1030H-Ag-82%-3 and 1030H-Ag-92%-3 was optimal when using DMF:PM (1:1) as solvent.

#### 3.3.2. Particle Size

**Figure 11:**
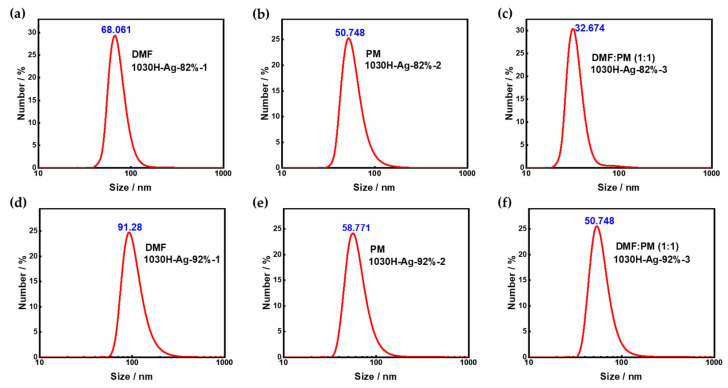
Particle size of silver conductive ink in different solvent systems. (**a**) 1030H-Ag-82%-1 conductive ink; (**b**) 1030H-Ag-82%-2 conductive ink; (**c**) 1030H-Ag-82%-3 conductive ink; (**d**) 1030H-Ag-92%-1 conductive ink; (**e**) 1030H-Ag-92%-2 conductive ink; (**f**) 1030H-Ag-92%-3 conductive ink.

The particle size of silver conductive ink is an important performance indicator that directly affects whether it meets printing requirements. Due to the tendency of nano silver particles to aggregate, the particle size of silver conductive ink can become too large, making it difficult for the conductive ink to pass through the fine nozzles of the printing head, which can cause printing problems such as residue, agglomeration, and unevenness, and even lead to nozzle clogging. Therefore, in order to improve printing quality and avoid printing faults, it is usually necessary to adjust the particle size of silver conductive ink. We tested the particle size distribution of 1030H-Ag-82% conductive ink and 1030H-Ag-92% conductive ink prepared with different solvents, and the results are shown in Figure 11.

As can be seen from the figure, under the same solvent conditions, the particle size of 1030H-Ag-82% conductive ink was lower than that of 1030H-Ag-92% conductive ink. The peak particle sizes of 1030H-Ag-82%-1 conductive ink and 1030H-Ag-92%-1 conductive ink were 68.06 nm (Figure 11a) and 91.28 nm (Figure 11d), respectively. This is mainly due to the tendency of nano silver particles to aggregate, resulting in a larger particle size of the conductive ink, while the 1030H resin can effectively inhibit the aggregation of silver nanoparticles and reduce the particle size of the conductive ink.

There are significant differences in the particle size distribution of conductive inks using different solvent systems. The particle size of the conductive ink prepared with DMF as solvent was larger, and the peak particle size of 1030H-Ag-92%-1 conductive ink was 91.28 nm (Figure 11d). The particle size of conductive ink fabricated with PM as solvent was medium, and the peak particle size of 1030H-Ag-92%-2 conductive ink was 58.77 nm (Figure 11e). The conductive ink using the mixed solvent system of DMF and PM had a lower particle size, and the peak particle size of 1030H-Ag-92%-3 conductive ink was 50.748 nm (Figure 11f).

Therefore, the DMF:PM (1:1) mixed solvent system is preferred for silver conductive ink.

#### 3.3.3. Conductivity

**Figure 12:**
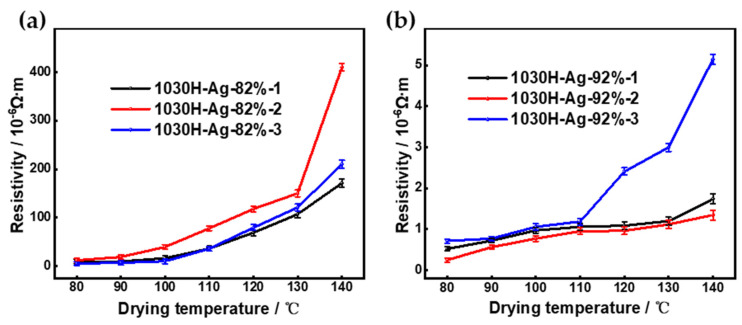
The resistivity of silver conductive ink prepared with different solvent systems at different drying temperatures. (**a**) 1030H-Ag-82% conductive ink; (**b**) 1030H-Ag-92% conductive ink.

The conductivity of printed patterns is an important indicator of conductive ink. Silver conductive ink was printed on a PET film, dried at different temperatures to achieve semi-curing by evaporating the solvent, and then hot-pressed at a pressure of 1 MPa and a temperature of 145 °C for 5 min. Then, it was heated in an oven from 120 °C to 160 °C at a rate of 10 °C/15 min and maintained at 160 °C for 1 h to obtain the printed pattern. We studied the effect of drying temperatures on the conductive properties of printed patterns, and the results are shown in Figure 12.

Figure 12a shows the resistivity of 1030H-Ag-82% conductive ink using different solvents at different drying temperatures. The change trend of conductive inks prepared with different solvent systems is consistent, and the resistivity increased with the increase in drying temperature. When the drying temperature increased from 80 °C to 140 °C, the resistivity of 1030H-Ag-82%-1 prepared with DMF increased from 9.16 × 10^−6^ Ω·m to 171 × 10^−6^ Ω·m, the resistivity of 1030H-Ag-82%-2 prepared with PM increased from 11.8 × 10^−6^ Ω·m to 410 × 10^−6^ Ω·m, and the resistivity of 1030H-Ag-82%-3 prepared with DMF:PM (1:1) mixed solvent increased from 5.27 × 10^−6^ Ω·m to 210 × 10^−6^ Ω·m. The high drying temperature accelerates the volatilization rate of the solvent, leaving air holes on the printed pattern, which increases the distance between the silver nanoparticles and reduces the conductivity of the silver conductive ink. However, the low drying temperature slows the volatilization rate of the solvent, which doubles the drying time. So, the optimal drying temperature was 90 °C. At a drying temperature of 90 °C, the conductivity of 1030H-Ag-82%-3 conductive ink (6.87 × 10^−6^ Ω·m) was better than that of 1030H-Ag-82%-1 conductive ink (9.55 × 10^−6^ Ω·m) and 1030H-Ag-82%-2 conductive ink (18.9 × 10^−6^ Ω·m).

Figure 12b shows the resistivity of 1030H-Ag-92% conductive ink prepared with different solvents at different drying temperatures. When the drying temperature increased from 80 °C to 140 °C, the resistivity of 1030H-Ag-92%-1 prepared with DMF increased from 0.524 × 10^−6^ Ω·m to 1.74 × 10^−6^ Ω·m, the resistivity of 1030H-Ag-92%-2 prepared with PM increased from 0.716 × 10^−6^ Ω·m to 5.15 × 10^−6^ Ω·m, and 1030H-Ag-92%-3 prepared with DMF:PM (1:1) increased from 0.253 × 10^−6^ Ω·m to 1.35 × 10^−6^ Ω·m. The conductivity of 1030H-Ag-92%-3 conductive ink (0.564 × 10^−6^ Ω·m) was better than that of 1030H-Ag-92%-1 conductive ink (0.724 × 10^−6^ Ω·m) and 1030H-Ag-92%-2 conductive ink (0.774 × 10^−6^ Ω·m) under the same conditions, at a drying temperature of 90 °C.

## 4. Conclusions

In summary, the resin binder of silver conductive ink (1030H silicone resin) was prepared with methylphenylamino silicone oil and epoxy-modified silicone oil that were successfully synthesized through functional silicone monomers. Additionally, low-temperature curing silver conductive inks were prepared with silver nanoparticles as conductive fillers, 1030H silicone resin as the resin binder, and DMF, PM, or DMF:PM (1:1) as solvent. The low-temperature curing silver conductive inks with DMF:PM (1:1) as solvent exhibited desirable dispersibility with particle sizes of 50–100 nm, good storage stability, and excellent adhesion. The printing performance and conductivity of the silver conductive ink prepared with DMF:PM (1:1) as solvent were better than those of the silver conductive ink prepared by DMF and PM solvent. Cured at a low temperature of 160 °C, the resistivity of low-temperature curing silver conductive inks with DMF:PM (1:1) as solvent was 0.564 × 10^−6^ Ω·m, so the low-temperature curing silver conductive ink has high conductivity. Compared with silver commercial conductive inks, our silver conductive inks had a lower curing temperature and higher adhesion. This research provides new insights for preparing low-temperature curing silver conductive ink, and hence will encourage more research studies to achieve extended practical applications.

## Figures and Tables

**Table 1:** Formulations and performance of different silver conductive ink.

Conductive Ink	Nano Silver (wt%)	1030H Silicone Resin (wt%)	DMF (wt%)	Adhesion Result	Pencil Hardness	Resistivity (×10^−6^ Ω·m)
1030H-Ag-78%	45.05	12.71	42.25	5B	3H	934
1030H-Ag-80%	46.36	11.59	42.05	5B	3H	591
1030H-Ag-81%	47.02	11.03	41.95	5B	3H	104
1030H-Ag-82%	47.68	10.47	41.85	5B	3H	28.10
1030H-Ag-84%	48.11	9.16	42.73	5B	2H	21.50
1030H-Ag-86%	50.35	8.20	41.45	5B	2H	9.42
1030H-Ag-88%	50.98	6.95	42.07	5B	2H	4.82
1030H-Ag-90%	52.44	5.83	41.74	4B	2H	1.73
1030H-Ag-92%	53.92	4.69	41.40	4B	2H	1.43

**Table 2:** Formulations of silver conductive inks with different solvent systems.

Conductive Ink	Nano Silver (wt%)	1030H Silicone Resin (wt%)	Solvent (wt%)	Solvent
1030H-Ag-82%-1	47.68	10.47	41.85	DMF
1030H-Ag-82%-2	47.68	10.47	41.85	PM
1030H-Ag-82%-3	47.68	10.47	41.85	DMF:PM (1:1)
1030H-Ag-92%-1	53.92	4.69	41.40	DMF
1030H-Ag-92%-2	53.92	4.69	41.40	PM
1030H-Ag-92%-3	53.92	4.69	41.40	DMF:PM (1:1)

## Data Availability

The data presented in this study are available upon request from the corresponding author.
